# High nutritional knowledge during adolescence is associated with healthier dietary habits in adulthood: a longitudinal cohort study

**DOI:** 10.1007/s00394-026-03968-y

**Published:** 2026-06-02

**Authors:** Laurent Béghin, Jules Morcel, Nathalie Michels, Mélanie Leroy, Camille Ternynck, Julien Labreuche, Stefaan De Henauwn, Maria L. Miguel-Berges, Angela Polito, Luis A. Moreno, Frédéric Gottrand

**Affiliations:** 1 Univ. Lille, Inserm, CHU Lille, U1286 - INFINITE –Institute for Translational Research in Inflammation, F-59000 Lille, France; 2 Department of Public Health, Faculty of Medicine and Health Sciences, Ghent University, Ghent, Belgium; 3 Department of Biostatistics, CHU Lille, F-59000 Lille, France; 4 Growth, Exercise, Nutrition and Development (GENUD) Research Group, University of Zaragoza, Saragossa, Spain; 5 Consorcio CIBER Fisiopatología de La Obesidad y Nutrición (CIBEROBN), Instituto de Salud Carlos III (ISCIII), Madrid, Spain; 6 Council for Agricultural Research and Economics– Research Center for Food and Nutrition – (formerly INRAN), Rome, Italy; 7 CIC-1403-Inserm-CHU de Lille Centre d’Investigation Clinique, Antenne Pédiatrique, Hôpital Jeanne de Flandre CHU de Lille, F-59000 Lille, France

**Keywords:** Nutritional knowledge, Diet composition, Adolescents, Young adults, Cardiovascular risk

## Abstract

**Purpose:**

Cardiovascular disease is the leading cause of mortality worldwide and is driven by atherosclerosis, which develops from a young age. Several lifestyle factors, including diet, physical activity, physical fitness, and sleep quality, influence cardiovascular health. However, limited research has investigated the association of nutritional knowledge with long-term health outcomes. This study assessed the association between adolescents’ nutritional knowledge and adulthood dietary habits, physical fitness, and cardiovascular risk factors.

**Methods:**

This longitudinal cohort study included 143 adolescents aged 12.5–17.5 years who were reevaluated as young adults (22–31 years) after a follow-up period of 10–14 years. The study was conducted in four European centers: Ghent (Belgium), Lille (France), Rome (Italy), and Zaragoza (Spain). Nutritional knowledge was assessed during adolescence and adulthood using the Nutritional Knowledge Test. Cardiovascular health was evaluated based on HDL and non-HDL cholesterol levels, blood pressure, body mass index, glycemia, and calculation of modified Pathobiological Determinants for Atherosclerosis in Youth (mPDAY) cardiovascular risk scores. Diet Quality Index (DQI), Planetary Health Diet Index (PHDI), ultra-processed food consumption, cardiorespiratory fitness (CRF) and upper-body muscular strength were also assessed. Spearman correlational analysis was used to identify associations between adolescents’ nutritional knowledge and cardiovascular, dietary, and physical fitness parameters in adulthood.

**Results:**

Correlation analysis showed that greater nutritional knowledge during adolescence was associated with a better dietary quality in adulthood, as reflected by a higher DQI (*p* = 0.004; r = 0.25; 95% CI [0.08–0.40]).

Nutritional knowledge during adolescence correlated inversely with the mPDAY cardiovascular risk score in adulthood. Cross-sectional analysis in adolescence and adulthood showed that nutritional knowledge in adolescence was not associated with cardiovascular parameters, physical fitness, or dietary quality indices during adolescence. Nutritional knowledge in adulthood was associated only with a better CRF (*p* = 0.003; r = 0.37 [0.13–0.58]) in adulthood.

**Conclusion:**

Nutritional knowledge during adolescence is associated with a dietary quality in adulthood, as measured by the DQI. These findings suggest that assessment of nutritional knowledge during adolescence might serve as a simple and effective tool for early identification of individuals at risk of poor dietary habits and increased cardiovascular risk. These findings also highlight the potential value of school-based interventions involving nutritional knowledge in promoting long-term cardiovascular health.

*NCT number*: NCT02899416

## Introduction

Cardiovascular disease originates in early life and is driven by the development of atherosclerosis, which is promoted by modifiable risk factors such as elevated body mass index (BMI), dyslipidemia, hypertension, hyperglycemia, and tobacco use [1, 2]. Although numerous short-term interventional and observational studies have highlighted the role of dietary intake, physical activity, and physical fitness as predictors of future cardiovascular health, longitudinal studies remain limited [3, 4]. Few nutritional studies have considered nutritional knowledge (NK) as a key determinant of cardiovascular health. Previous research has established associations between greater NK and greater adherence to the Mediterranean diet [5] or improved dietary habits [6]. We previously reported an association between NK in adolescence and lower cardiovascular risk in adulthood [7] and the underlying determinants remain poorly understood. The present study aimed to identify the nutritional and physical parameters linking dietary knowledge in adolescence to future cardiovascular risk reduction in adulthood.

## Materials & methods

### Study population

This study used data from the Healthy Lifestyle in Europe by Nutrition in Adolescence (HELENA) study, which collected information about lifestyle parameters—including nutrition, physical activity, physical fitness, anthropometric measures, and biological samples—from European adolescents in 10 European countries [8]. A decade later, the Better Life by Nutrition During Adulthood study followed up with these participants. The current analysis included 143 participants with complete data sets. The study was conducted across four European centers: Ghent (Belgium), Lille (France), Rome (Italy), and Zaragoza (Spain). There were no restrictive inclusion criteria, only that participants must have participated in the HELENA study, signed an informed consent form, and had health insurance at the time of enrollment. This study had been carried out in accordance with the Declaration of Helsinki. Further details on the study population and methodology have been published elsewhere [7, 9].

### Study design

Participants were reassessed at ages 21–32 years, which was 10–14 years after their initial participation. The follow-up visit included assessment of anthropometric measures, physical activity, and fitness, completion of questionnaires about NK and dietary habits, and collection of biological samples. Standardized tools and materials ensured comparability between the adolescent and adult assessments. The primary analysis examined the relationships between adolescent NK and cardiovascular risk, dietary habits, and physical fitness in adulthood. Cross-sectional associations between NK, dietary quality indices, and cardiovascular risk factors were also assessed at both time points.

### Data collection

Dietary intake was evaluated using three nonconsecutive 24-h dietary recall surveys. Nutritional parameters included ultra-processed food (UPF) consumption, classified using the Nova classification system [10, 11], adherence to the Diet Quality Index (DQI) [12], and adherence to the Planetary Health Diet Index (PHDI) [13]. The DQI is a semiquantitative food frequency questionnaire validated according to the Flemish food-based dietary guidelines and based on dietary quality, diversity, equilibrium, and meal patterns [12]. The PHDI is also a food frequency questionnaire based on the recommendations of the reference diet proposed by the EAT-Lancet commission [13]. NK was assessed using the validated Nutritional Knowledge Test (NKT) a 23-item multiple-choice questionnaire [14–16]. This questionnaire covers key domains of general nutrition knowledge, including dietary recommendations, nutrient sources, and diet–health relationships. All items were presented in a standardized order and completed under the supervision of trained study staff to ensure consistency in administration. This questionnaire was scored as follow: good answer given 1 point and incorrect answer given 0 point. Participants were given a score out of 23 questions, converted into a percentage of correct answer for analysis. Missing responses were treated as incorrect answers in the scoring procedure. Higher scores reflected a greater level of nutritional knowledge and were analyzed as a continuous variable. The NKT was administered during 15 min sessions at school for adolescents and in a clinical/hospital setting for adults. The same version of Sichert-Heller et al. [16] was used for adolescents and adulthood population.

Participants who completed < 75% of the NKT were excluded from the analysis.

The cardiovascular risk factors analyzed in adulthood included high-density lipoprotein (HDL) cholesterol, non-HDL cholesterol, and glycated hemoglobin (HbA1c) levels, mean blood pressure (MBP), systolic blood pressure (SBP), diastolic blood pressure (DBP), and BMI (kg/m^2^). MBP was calculated as (SBP + 2 × DBP) / 3. We also used modified Pathobiological Determinants for Atherosclerosis in Youth for Coronary Arteries and Abdominal Aorta (mPDAY CA and AA, respectively) scores to assess future cardiovascular risk. The details of the calculation method have been reported elsewhere [2, 7]. At baseline, only 1/3 of HELENA adolescents were assessed for cardiometabolic variables (e.g., HDL and non-HDL cholesterol) according to the HELENA study protocol [publi 9].

Physical fitness parameters included upper-body muscular strength (UBMS), measured using a handgrip test, and cardiorespiratory fitness (CRF), assessed using the 20-m shuttle run test [17]. These parameters have been shown to have predictive value for adult cardiovascular risk in the same cohort [7].

### Statistical analysis

Categorical variables are described in terms of numbers and percentages. Non-Gaussian quantitative variables are reported as median and interquartile range (IQR) and Gaussian quantitative variables as mean and standard deviation. The normality of the distributions was checked graphically and tested using the Shapiro–Wilk test. Correlations of quantitative variables in adolescence with the NKT in adolescence were assessed using Spearman correlational analysis after adjustment for age, gender and socio-economic status. Correlations of the same quantitative variables and NKT during adolescence and adulthood were also assessed. All results are expressed as adjusted Spearman’s correlation coefficients and their 95% confidence intervals (CIs). No multiple imputation was applied. Statistical tests were performed at the two-tailed α-level of 0.05 using SAS software (version 9.4).

## Results

**Fig. 1:**
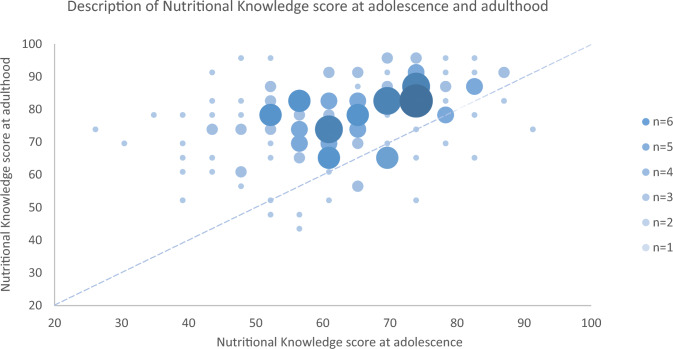
Scatter bubble plot descripting the Nutritional Knowledge score at adolescence and adulthood. Data expressed as percent of right answers. The size of the bubble corresponds to the sample with same scores in adolescence and adulthood. The data above the blue dotted line correspond to subjects that progressed between adolescence and adulthood. The data below correspond to individuals who have regressed

The study population has been described previously and is characterized by a relatively high education level, low prevalence of overweight/obesity (21%), and no reported cardiovascular events [7, 9]. The final analysis included 143 participants with available data. The descriptive characteristics for the adolescents and adults are presented in Table 1. Only a few of the variables changed between the time points: UBMS, UPF intake, and NKT score. HbA1c level was not measured during adolescence. The NKT score improved in most of the study population between adolescence and adulthood (Fig. 1).

**Table 1:** Characteristics of the subjects in adolescence and adulthood

	n	Adolescents	n	Adults
Age (*years*)	143	14.9 ± 1.1	143	25.5 ± 1.2
Gender, women *(%)*	143	52.4	143	52.4
*Cardiovascular*				
BMI (*kg/m*^*2*^)	143	20.0 [18.3–21.6]	143	22.3 [20.9–24.6]
HDL* (*mg/dL*)	51	57.0 [49.0–65.0]	143	60.0 [51.0–72.0]
Non-HDL* (*mg/dL*)	51	100.8 [89.1–111.2]	143	105.0 [87.0–132.0]
HbA1c** *(%)*	–	–	143	5.2 [5.0–5.3]
SBP (*mmHg*)	143	117.0 [109.0–127.0]	143	115.0 [107.0–124.0]
DBP (*mmHg*)	143	66.0 [62.0–70.0]	143	68.0 [62.0–73.0]
MBP (*mmHg*)	143	83.7 [70.3–117.3]	143	83.7 [78.3–89.0]
mPDAY CA** (from − 1 to 25)	–	–	143	0 [ − 1–1]
mPDAY AA** (from 0 to 14)	–	–	143	0 [ − 1–4]
*Nutrition*				
DQI (*from* − *33 to 100*)	115	66.7 [57.7–74.7]	136	66.0 [60.0–74.0]
PHDI (*from 0 to 150)*)	114	46.7 ± 12.6	126	49.8 ± 10.7
UPF *(% of total daily energy intake*)	126	58.7 [47.6–67.2]	118	39.9 [25.3–53.7]
Fitness				
NKT (*% of right answers*)	143	60.9 [52.2–73.9]	143	78.3 [69.6–87.0]
UBMS (*kg*)	143	28.4 [24.2–35.2]	143	36.3 [34.0–49.1]
CRF (*level*)	131	6.0 [5.0–8.0]	61	6.0 [4.0–8.0]

*Data collected on 1/3 of adolescent subject

**Data only measured in adulthood

For adolescents and adults, values are presented as mean ± standard deviation or median [inter quartile range]

*BMI* Body mass index; *CRF* Cardiorespiratory fitness; *DBP* Diastolic Blood Pressure; *DQI* Diet Quality Index; *HbA1c* Glycated Haemoglobin; *HDL* high-density lipoprotein; *MBP* Mean blood pressure; *NKT* Nutritional Knowledge Test; *PDAY AA* PDAY risk score for abdominal aorta; *PDAY CA* PDAY risk score for coronary arteries; *PHDI* Planetary Health Diet Index; *SBP* Systolic Blood Pressure; *UMBS* Upper body muscular strength; *UPF* Ultra-processed food

**Fig. 2:**
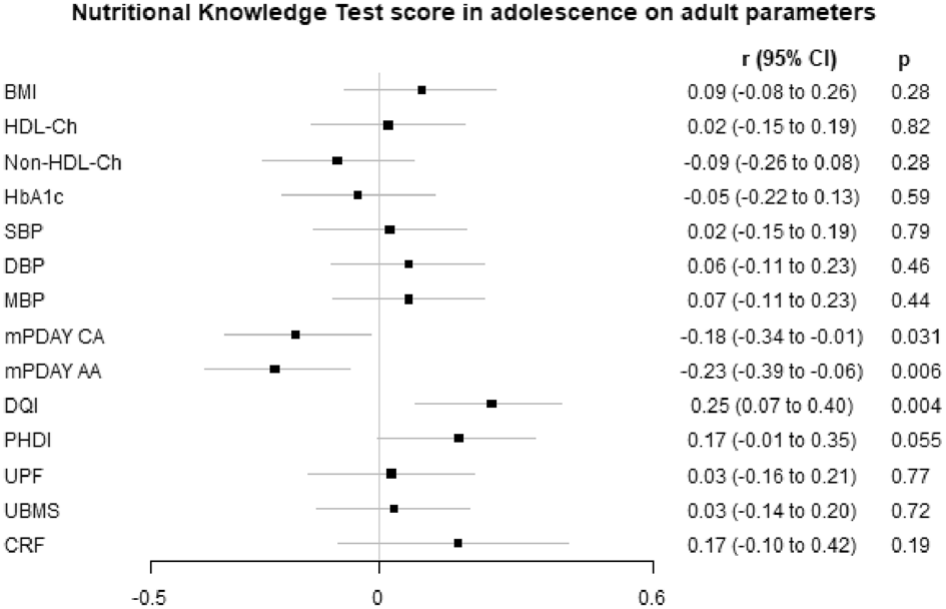
Correlation of NKT score in adolescence and the interest parameters in adulthood. Results are expressed as Spearman’s correlation coefficients (95% CI) with adjustment for age and gender and, as adjusted P value. BMI, body mass index; CRF, cardiorespiratory fitness; DBP, Diastolic Blood Pressure; DQI, Diet Quality Index; HbA1c, Glycated Haemoglobin; HDL, high-density lipoprotein; MBP, mean blood pressure; NKT, Nutritional Knowledge Test; PDAY AA, PDAY risk score for abdominal aorta; PDAY CA, PDAY risk score for coronary arteries; PHDI, Planetary Health Diet Index; SBP, Systolic Blood Pressure; UMBS, upper body muscular strength; UPF, ultra-processed food

**Fig. 3:**
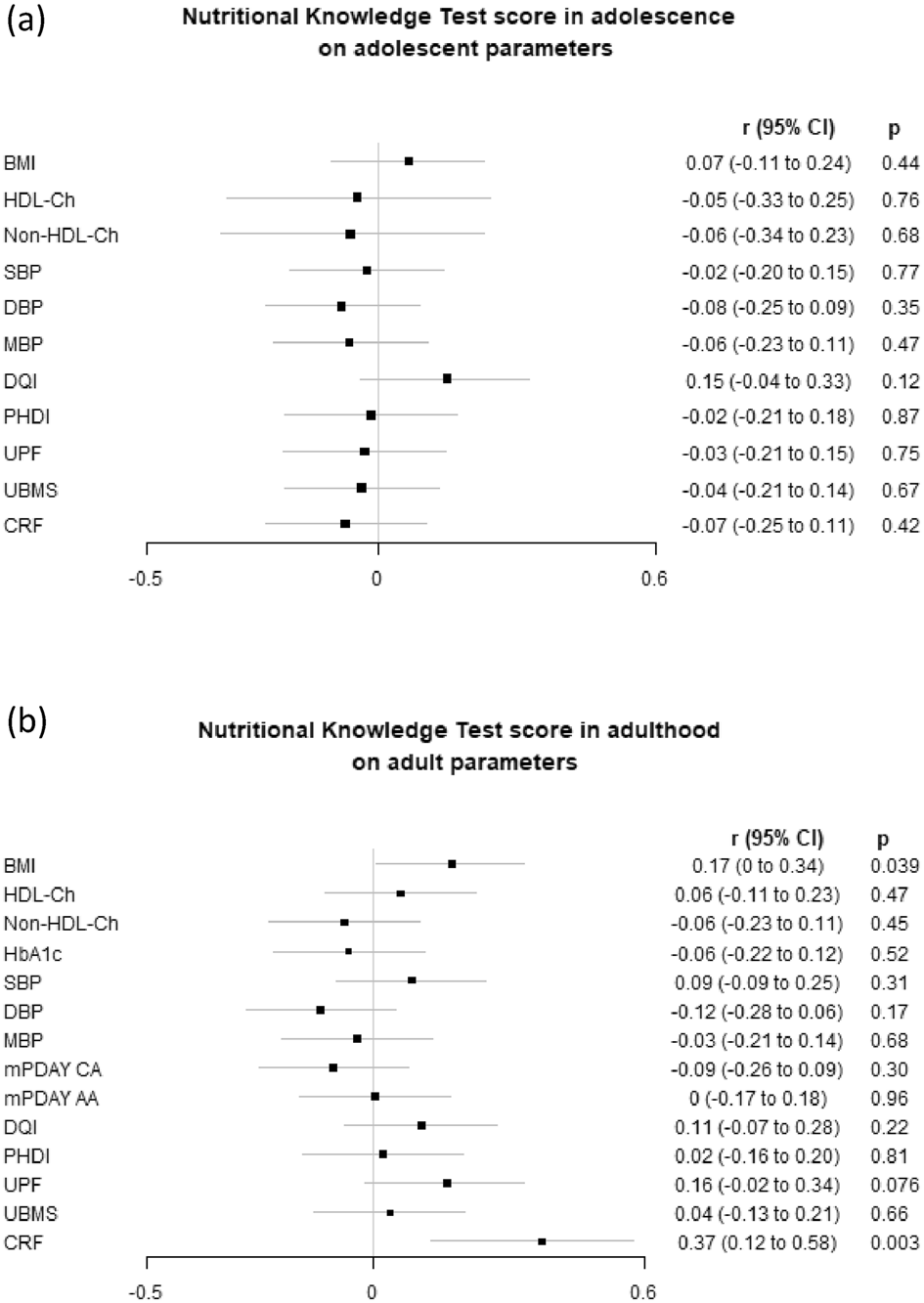
Correlation of NKT score in adolescence and the interest parameters in adolescence (**a**) and correlation of NKT score in adulthood and the interest parameters in adulthood (**b**). Results are expressed as Spearman’s correlation coefficients (95% CI) with adjustment for age and gender and, as adjusted P value. BMI, body mass index; CRF, cardiorespiratory fitness; DBP, Diastolic Blood Pressure; DQI, Diet Quality Index; HbA1c, Glycated Haemoglobin; HDL, high-density lipoprotein; MBP, mean blood pressure; NKT, Nutritional Knowledge Test; PDAY AA, PDAY risk score for abdominal aorta; PDAY CA, PDAY risk score for coronary arteries; PHDI, Planetary Health Diet Index; SBP, Systolic Blood Pressure; UMBS, upper body muscular strength; UPF, ultra-processed food

A positive association was observed between the adolescent NKT score and dietary quality in adulthood (Fig. 2). This was reflected by a higher DQI (*p* = 0.004; r = 0.25 [95% CI 0.08–0.40]) and lower mPDAY CA and AA scores (*p* = 0.031; r = –0.18 [95% CI –0.34 to –0.02] and *p* = 0.007; r = –0.23 [95% CI –0.38 to –0.06], respectively). The forest plot presented in Fig. 3A shows that the adolescent NKT score did not correlate with any parameters of interest in adolescence. The forest plot in Fig. 3B shows that higher NKT score in adulthood correlated significantly with higher in CRF adulthood (*p* = 0.003; r = 0.37 [95% CI 0.13–0.58]).

## Discussion

To our knowledge, this is the first study to assess longitudinally the relationship between NK and dietary habits, physical fitness, and cardiovascular risk over time. Previous research has linked NK to body weight [18, 19] and dietary behaviors [20–25], but these studies were cross-sectional and did not examine long-term associations. Given that adolescence represents a critical period for acquiring autonomy in food choices, understanding the effects of NK on long-term cardiovascular health is essential.

Our findings indicate that high NK in adolescence is associated with improved dietary quality in adulthood and confirm that NK is inversely related to cardiovascular risk as measured by mPDAY risk scores. This association was checked by recalculating the correlation coefficients after removing 9 individuals with extreme change (changes < -10 and 5 individuals with changes > 40). The exclusion of these individuals does not alter the results (data not shown). However, we found no significant correlations between adolescent NK and any single cardiovascular or dietary parameter in adolescence. Interestingly, adult NK was nearly, but not significantly, associated with healthier dietary behaviors, which may explain the absence of a direct link between NK and lower cardiovascular risk in adulthood. Although the inverse correlation between NK and the mPDAY score reached statistical significance, the strength of the association (r =  − 0.21) was relatively modest. This suggests that higher levels of NK are associated with a slightly lower predicted cardiovascular risk in early adulthood. However, the magnitude of the correlation indicates that NK explains only a small proportion of the variability in mPDAY scores. While modest at the individual level, such associations may still be meaningful at the population level, particularly when the exposure is common and operates alongside other determinants of cardiovascular risk.

Excepted for CRF, no significant associations were observed between adult NK and cardiovascular, PF, or dietary parameters, which suggests that the relationships we found between NK and outcomes in adulthood originated from adolescence and were not directly influenced by the actual NK level in adulthood. A plausible explanation for the observed association between NK and CRF in adulthood relates to overall diet quality. Individuals with higher levels of NK are more likely to consume greater amounts of fruits and vegetables, resulting in higher intakes of key micronutrients, particularly Vitamin C [24, 25]. Vitamin C plays a role in endothelial function, oxidative stress regulation, and aerobic mitochondrial metabolism which may influence CRF. Higher circulating concentrations of Vitamin C could therefore contribute, at least partially, to be associated with high levels of CRF [26]. From this perspective, diet quality may act as a partial mediator in the relationship between NK and CRF. In contrast, other components of PF especially UBMS, which is primarily supported by anaerobic energy pathways, is not influenced and may depend more strongly on specific determinants including resistance training, muscle mass, and genetic factors [27]. This may explain the absence of similar associations for UBMS in this study.

Our previous analysis demonstrated that DQI and PHDI are linked to lower cardiovascular risk [7], which reinforces the importance of identifying strategies to improve these indices since adolescence. These findings suggest that NK has a stronger long-term predictive value than immediate dietary behavior and may clarify the inconsistent results of previous cross-sectional studies [16, 23].

We found no significant associations between adolescent nutritional knowledge, food quality, and any of the physical fitness parameters and cardiovascular risk factors at the same age category. This lack of associations suggests that knowledge does not translate immediately into feeding comportment and health benefits during adolescence, but probably needs time to be more anchored in daily life and lead gradually to better nutritional habits and improved cardiovascular health. Improving NK as early as possible might be a preventive tool and worthwhile investment for future health. According to our results, adults with the best dietary knowledge are likely to see their diet and health parameters improve in the future. The significant associations between nutritional knowledge and food quality in adolescence and health parameters in adulthood support the rationale for nutritional education during adolescence, as well as the potential for this education to yield delayed but significant health benefits.

The study’s strengths include its longitudinal design, consistent measurement methodologies across time points, and relatively homogeneous study population, which reduced the potential for bias for interpretation of the results. The study’s limitations include (*i*) a selection bias of the entire BELINDA population. The differences found between HELENA subjects who were participants or non-participants in the BELINDA study were only shown for BMI and maternal educational level [9]. In addition, the absence of baseline cardiometabolic variables (e.g., glycemia, HDL and non-HDL cholesterol) of 2/3 of the HELENA adolescents could led for a second type of selection bias. A Comparison of adolescents with or without baseline cardiometabolic blood measures was performed for gender, age, BMI and socio-economic status of the mother and did not found any difference (data not shown). (*ii*) A small sample size at follow-up (n = 143) not allowed multivariate analyses and interaction testing. (*iii*) An absence of genetic/family history characteristics. (*iiii*) The use of self-reported dietary data which could make some recall bias/misclassification. Although these findings suggest robust associations between adolescent NK and adult dietary and health parameters, causality cannot be inferred given the noninterventional nature of the study.

The findings of the present study underscore the long-term importance of NK in shaping future dietary habits and reducing cardiovascular risk. The NKT is a simple, effective tool for identifying populations at risk of poor dietary habits and future cardiovascular disease. It may also serve as a valuable instrument for school-based interventions and preventive health strategies.

## Data Availability

Not applicable.
